# Case report of rapidly progressive proliferative verrucous leukoplakia and a proposal for aetiology in mainland China

**DOI:** 10.1186/1477-7819-9-26

**Published:** 2011-02-27

**Authors:** Lin Ge, Yun Wu, Lan-yan Wu, Lin Zhang, Bing Xie, Xin Zeng, Mei Lin, Hong-mei Zhou

**Affiliations:** 1 State Key Laboratory of Oral Diseases, Sichuan University, Chengdu, Sichuan, PR. China; 2 Department of Oral Pathology, West China College of Stomatology, Sichuan University, Chengdu, Sichuan, PR. China; 3 Department of Oral Medicine, West China Hospital of Stomatology, Sichuan University, Chengdu, Sichuan, China

## Abstract

Proliferative verrucous leukoplakia (PVL) is a rare oral leukoplakia and has four features such as chronic proliferation, multiple occurrences, refractoriness to treatment and high rate of malignant transformation. As mentioned above, most PVL cases processed to malignancy over many years, sometimes 20 years. However, this report described a case of rapid progress, which had malignant transformation in a short period. Additionally, the aetiology of PVL was discussed and immunity was proposed as the possible cause.

## Introduction

Proliferative verrucous leukoplakia (PVL) is a rare oral leukoplakia, principally characterized by chronic proliferation, multiple occurrences, and refractoriness to treatment. Its rate of malignant transformation is extremely high [1]. The characteristics of its clinical and pathological progress are considered vital bases for the diagnosis of PVL because there are no particular differences between the pathological changes of PVL and those of oral verrucous leukoplakia (OVL) [2].

PVL grows slowly and can take up to 7.8 years to become cancerous. The process is irreversible and usually progresses to cancer. According to the study by Bagan, PVL quickly becomes malignant, on average within 4.7 years [3], whereas Hansen reported an average time to cancer of 6.1 years [1]. However, Silverman and Gorsky reported a longer mean malignant process of 11.6 years [4].

Recently, our department treated a patient with PVL that developed extremely rapidly, with only 16 months from the appearance of white patches to their cancerous transformation. Consequently, this case warrants attention. We describe this case with reference to the relevant literature, and confirm that this is the first report of PVL in mainland China.

## Case report

A female patient, aged 52 years, attended the Department of Oral Medicine at West China Hospital of Stomatology, Sichuan University in June, 2006, with painless white patches over the right bucca and palate for over a year. One year earlier, the patient had discovered the white patches on her right bucca and palate, which felt coarse but were painless. The local hospital diagnosed them by biopsy as leukoplakia, but did not treat them.

**Figure 1:**
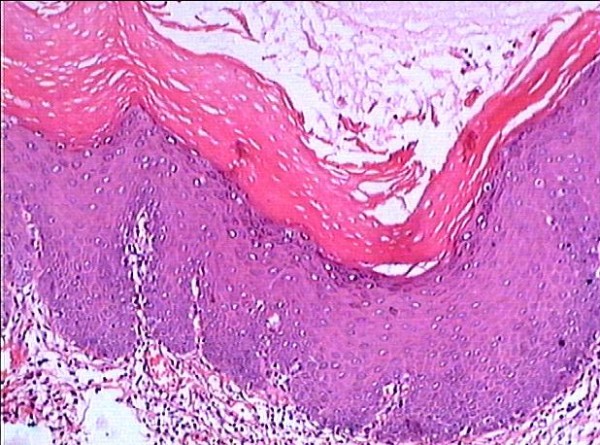
**The right buccal verrucous leukoplakia with mild to moderate dysplasia(1^st ^biopsy, HE, original magnification × 100)**.

**Figure 2:**
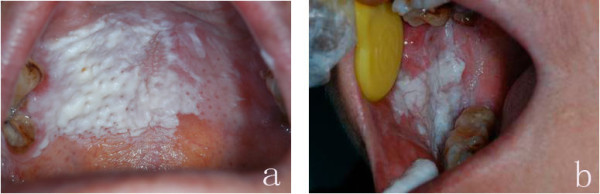
**A broad white patch was seen on the right of palatal mucosa, its surface was prominent like multiple peaks (a); The white patch over the right bucca was obvious thicker and extra-salient (b)**.

The patient came to our hospital as the situation worsened. On a physical examination, her face was symmetrical and not swollen. Extensive white lesions, with multiple peaks on their surfaces, were seen over the right bucca, which were coarse and tough on palpation, but with no congestion or erosion. A white patch like crepe paper was apparent on the C5-7 buccal gingiva and vestibular sulcus. An even white patch, with a soft mucosal texture was present on the left buccal mucosa, along the line of occlusion. White patches occurred from the palatal gingiva, close to A6-7, to the midline. Some white patches, similar in size to rice grains or soybeans, appeared over the lingual rim on both sides and the dorsum. A biopsy of the most affected part of the right bucca showed that the condition was verrucous leukoplakia with mild to moderate dysplasia (Figure 1). By combining the characteristics of the oral lesions and the pathological changes, a primary diagnosis was drawn of either OVL or PVL. Because the patient rejected the surgery proposed by a maxillofacial surgeon combined with P53 biotreatment, we proceeded as follows: 1) an overall physical examination was suggested to exclude any hidden malignant tumour; 2) the patient's immunity was enhanced, and retinoic acid and nystatin were given as topical therapy; 3) close surveillance was undertaken, with periodic checks upon request. The physical examination revealed that the patient only suffered from chronic superficial antral gastritis, and no malignant tumour was found elsewhere in her body. During the first examination on July 31, 2006 (one month after treatment), the patient said that the lesions were slightly relieved by the medication. A physical examination showed no obvious changes in the white patches over the right bucca and tongue. However, extensive white patches with rough and uneven surfaces were still visible from the C5-7 buccal gingiva to the vestibular sulcus and on the C7 disto-gingiva, which had become much more conspicuous since her first visit. Because the white patch on the right side of the palate had become thinner and smaller, the therapeutic regimen was continued. On the physical examination at the patient's second visit on August 30, 2006 (two months after treatment), a white patch was obvious on the right side of the palate, which was tough in texture, prominent over the mucosa, coarse and without tenderness. The white patches on the right bucca, C5-7 gingiva, left bucca, and tongue had not changed. As well as strengthening the patient's immunity and the topical application of retinoic acid, fluconazole paste was added to the treatment regimen. When the patient was examined for the third time on October 18, 2006 (about four months after her initial treatment; she had run out of retinoic acid two weeks earlier because she had delayed this examination), the white patch on the right bucca was markedly thicker, especially prominent, tough, and enlarged. Thickened white patches were visible on the C5-7 buccal gingiva and the C6-7 lingual gingival. A broad white patch was present on the palatal mucosa opposite A5-7, the surface of which was raised, with multiple peaks and a hard texture extending over the midline and close to the gingiva on the opposite side. The palatal lesions had clearly worsened, although there was no notable change in the white patches on the left bucca or tongue (Figure 2). Therefore, the diagnosis was revised to PVL (malignant transformation suspected), consistent with the characteristics of the lesions, the therapeutic reaction, and the progress of the disease.

**Figure 3:**
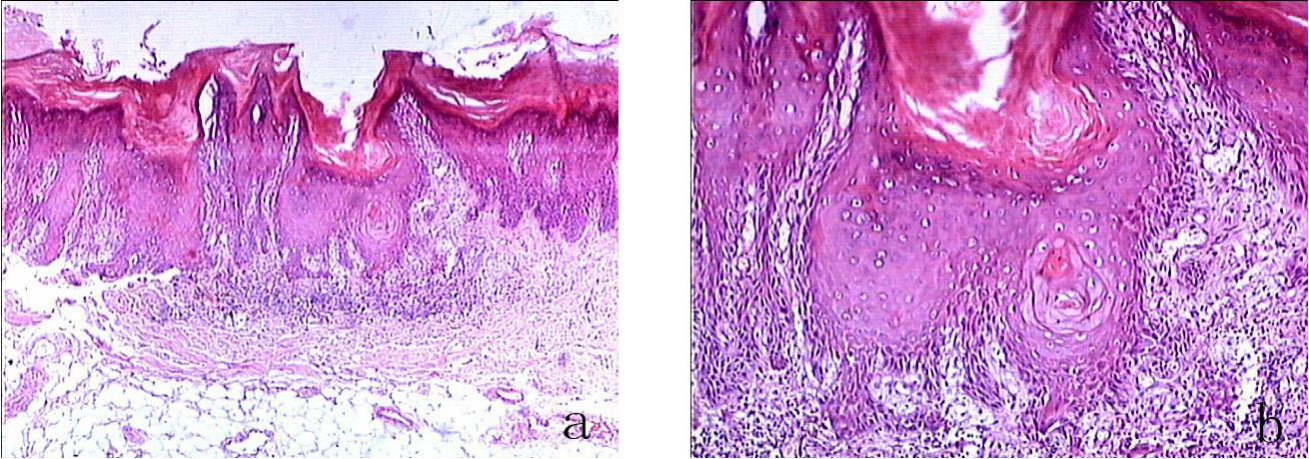
**The palatal carcinoma in situ was mildly invasive (a: HE, original magnification × 40, b: HE, original magnification × 100)**.

**Figure 4:**
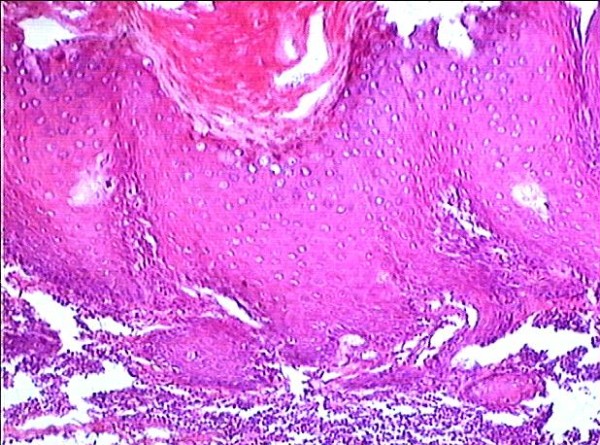
**The right buccal verrucous leukoplakia with moderate dysplasia( 2^nd ^biopsy, HE, original magnification × 100)**.

Because the patient's response to drug therapy was poor and the lesions had grown rapidly over the preceding four months, she was transferred, with her and her family's permission, to the Department of Oral and Maxillofacial Surgery for an operation to remove the white patches from the right side of the palate, bucca, and mandibular gum, and to simultaneously undergo tissue repair with skin grafting. The wound healed well after surgery. A histological examination revealed that the palatal carcinoma in situ was mildly invasive, and that the verrucous leukoplakia on the right bucca showed moderate dysplasia (Figure 3, Figure 4). The patient left hospital two weeks after surgery. Since then, she and her family have preferred palliative treatment. She has agreed to periodic examinations.

## Discussion

### General properties of PVL

PVL is a rare and specific disease that differs from OVL, and is often seen in middle-aged and elderly women, occurring predominantly on the bucca, palate, gingiva, and tongue. Hansen et al. [1] classified the pathological process of PVL into 10 grades, i.e., normal oral mucosa (0), homogeneous leukoplakia (2), verrucous hyperplasia (4), verrucous carcinoma (6), papillary squamous cell carcinoma (8), and poorly differentiated carcinoma (10), in which the odd scores refer to a status intermediate between those referred to by the adjacent even scores. Once PVL is confirmed, active therapy should be undertaken, such as surgery, laser management, photodynamic therapy, combined treatments, etc. [5-9]. However, PVL responds poorly to various therapeutic measures, and its recurrence rate is relatively high, even after its surgical removal.

### Developmental process of PVL and related epidemiological investigation in China

PVL is usually chronic and progressive, and a patient often has a long history of leukoplakia before he/she attends a clinic [8,10]. Most cases progress for 20-25 years. In contrast to most slow-growing PVL, the case described here became cancerous quite rapidly. 1) There was only a short history of leukoplakia; the duration of the disease preceding the patient's first visit was only one year, according to the patient. 2) There was no obvious growth process from single foci to multiple foci. 3) The lesions changed quickly; the disease was clearly more hyperplastic in the fourth month after the initial visit. 4) The period to malignancy was short; the whole process in this case took less than two years. The white patch over the palate was shown by biopsy to have undergone malignant transformation within about four months of the initial visit.

The disease reported here developed rapidly within four months of the patient's initial clinic visit. Therefore, we speculate that when PVL progresses to moderate dysplasia or malignancy, it is supposed to develop rapidly and not remain so chronic as its early stage. Furthermore, previous studies have primarily focused on Caucasian subjects, reflecting the growth status and properties of PVL only among these ethnic groups, so there is little knowledge of PVL in Asian or specifically Chinese populations [11]. Therefore, it must be determined whether PVL has different features in these populations.

China undertook an epidemiological census of oral leukoplakia in 134,492 people between 1978 and 1979. The results showed that 14,076 of the subjects had oral leukoplakia, 287 of whom had warty lesions, constituting a large proportion (68.33%) of the 420 patients with heterogeneous leukoplakia [12]. A longer observation period is required to establish a definite diagnosis of PVL, to allow its progression, because in its initial stages, PVL appears to be simple verrucous leukoplakia. Therefore, the incidence of PVL in China requires a long-term longitudinal study.

### Aetiology of PVL

Until now, the aetiological factors of PVL have been unclear. However, the case reported here and those in the literature seem to implicate immune factors. As reported, our patient suffered from chronic superficial antral gastritis, which would affect nutrient absorption and further affect the immunity of the patient. Enhancing the patient's immunity and topical therapies had a positive effect at the first examination. The report of a patient with PVL after bone-marrow transplantation (BMT) [13] supports this impression. BMT involves an immunosuppressive step and oral squamous cell carcinoma (OSCC) is a malignancy that can occur after BMT. This indicates that immunity plays an important role in PVL, as in OSCC. Epidemiological data have demonstrated that there is a high incidence of PVL in elderly women, with no obvious association with cigarette smoking and alcohol consumption, which distinguishes PVL from other ordinary leukoplakias. Common sense tells us that women have lower immunity than that of men and that immunity decreases with age. This implies that immune factors, rather than external stimuli, play a major role in PVL. Moreover, PVL patients infected with human papillomavirus [7,14] or Epstein-Barr virus [15] might be immunocompromised like human immunodeficiency virus -infected patients [16]. If immunity plays an important role in PVL, enhancing the immune response is a critical intervention, especially in the early phase of the disease because some patients have shown resistance to such therapies in later stages.

## Conclusions

Whether PVL progresses especially rapidly in Asian or Chinese populations requires further investigation. The health of these patients, especially their immune status, warrants examination for its contribution to the aetiology of PVL.

## Consent

Written informed consent was obtained from the patient for the publication of this case report and any accompanying images. A copy of her written consent is available for review by the Editor-in-Chief of this journal.

## Competing interests

The authors declare that they have no competing interests.

## Authors' contributions

GL and WY tracked the clinical data and drafted the manuscript. WL provided the pathological technique. HX and ZX participated in the design of the study. ML and HZ conceived of the study, and participated in its design and coordination and helped to draft the manuscript. All authors read and approved the final manuscript.
